# A behavioral typology of opioid overdose risk behaviors among recent veterans in New York City

**DOI:** 10.1371/journal.pone.0179054

**Published:** 2017-06-08

**Authors:** Alex S. Bennett, Andrew Golub, Luther Elliott

**Affiliations:** 1 National Development and Research Institutes Inc., New York, New York, United States of America; 2 Affiliated Investigator, Center for Drug Use and HIV/HCV Research, Rory Meyers College of Nursing, New York University, New York, New York, United States of America; Technion Israel Institute of Technology, ISRAEL

## Abstract

**Objective:**

To identify meaningful classes of opioid-using military veterans in terms of self-reported opioid overdose risk behaviors.

**Method:**

The study recruited a sample of 218 military veterans in the NYC area who were discharged from active duty service after September 11, 2001 and reported past-month opioid use. Survey data including measures of mental health, social stressors, substance use, and opioid-related overdose risk behaviors were analyzed using Latent Class Analysis (LCA).

**Results:**

A five group solution had excellent fit scores and interpretability. Factor analysis confirmed the existence of two major dimensions of variation: non-adherence and heroin use. The five groups included lower-risk prescription opioid users, non-adherent prescription opioid users and heroin users. The non-adherent prescription opioid users and heroin user classes were both further subdivided into “occasional” and “regular” use categories. In addition to endorsing a greater number of overdose risk behaviors, users in the regular use classes were more likely to screen positive for alcohol and substance use disorders, reported greater self-medicating opioid use to relieve anxiety, reported greater problems with physical pain, were more likely to have had mental health, alcohol and drug treatment, and were less likely to be employed or in school. Heroin users also were less likely to report stable housing.

**Conclusions:**

Findings indicate that opioid overdose risk classes are grounded in contextual factors related to experiences of psychological, physiological, and social adjustment pain and distress which should be addressed in tailored interventions targeting opioid users’ unique constellations of risk behaviors and comorbid conditions.

## Introduction

America is facing a health crisis of drug overdose driven by the use of prescription opioids (POs) and heroin. Since the late 1990s when patients’ rights to effective pain treatment became a mainstay of medical ideology in the US [1], POs have been widely prescribed to a broad cross-section of the population [2, 3], leading to markedly increased rates of both medical and nonmedical use. Consequently, from 1999 to 2013, the drug poisoning fatality rate more than doubled from 6.1 to 13.8 people per 100,000, and the rate of drug poisoning deaths involving opioid analgesics nearly quadrupled from 1.4 to 5.1 people per 100,000 [4].

Though PO use has remained relatively constant from 2007–2012 in the US, past year heroin use nearly doubled (373,000 to 669,000 users) in 2012 [5]. In New York City, where this study takes place, after a brief decline in the late-2000s, drug overdose deaths increased by 42% between 2010–2014 resulting in 797 drug overdose deaths in 2014 [6]. Of these drug overdose deaths, opioids were involved in 79%—POs were involved in 27%, and heroin was involved in 57% [6]. There are many possible explanations for overdose. One of them involves possible prescribing practices: patients who are prescribed POs sometimes are also prescribed other psychoactive drugs, such as benzodiazepines for anxiety, which can interact with POs to increase overdose incidence of morbidity and mortality [7–10]. Others involve not following instructions meticulously; for example, the use of alcohol should be avoided when taking opioids.

This epidemic has greatly impacted active-duty military personnel and veterans who have experienced high rates of opioid misuse and overdose [11–13]. A 2014 Veterans Administration (VA) report indicated that 440,000 patients were currently prescribed opioids, placing them at potential risk for overdose, and 55,000 patients were currently diagnosed as having an opioid use disorder, placing them at even greater risk [14].

This paper seeks to add to our understanding of risky behaviors among veterans taking opioids to inform future interventions that my prevent overdose. Overdose risk has traditionally been difficult to measure empirically. However, a robust body of research on opioid overdose risk behaviors [15–21] has provided a basis for understanding common OD risks, and several scales such as the Current Opioid Misuse Measure [COMM; 22, 23] and the Screener and Opioid Assessment for Patients with Pain [SOAPP; 24] have been shown to be robust and internally valid. The body of research on non-fatal overdose has provided important insights into a broad array of opioid-using populations, including both younger and older adults, people who inject drugs (PWID), the formerly incarcerated, and various professional populations [25–30]. This research has established behaviors associated with overdose, but there is a need for better tailored and targeted interventions and understandings of the ways in which certain sets of substance abuse behaviors are tied together—by psychological traits, market forces, sociocultural dimensions, or some combination thereof.

Latent class analysis (LCA) involving substance users has proved a valuable tool in identifying clinically and culturally meaningful subgroups, whether defined by abuse severity [31], types of substance and polysubstance use [32, 33], or comorbid substance use disorders and traumatic experiences or health conditions [34–36]. Several recent analyses of opioid users have established interpretable subgroups of PO users and misusers and their varying degrees of overdose risk [36] as well as important differences between PO users (many of whom appear to be self-medicating depression) and heroin users based on injection status, polysubstance use involving crack cocaine, and homelessness [37]. These findings demonstrate the complex contextual dimensions of significant typologies and suggest ways of targeting the nexus of risk factors that confront different user subpopulations.

In this paper, we examine typologies of opioid-related overdose risk behaviors among military veterans in NYC and identify the most common combinations of behaviors and their associations with physiological, psychological, and social pains and stresses.

## Methods

### Study sample and data collection

The “Opioid Misuse and Overdose Risk Patterns among Recent Veterans” study used targeted venue-based sampling and chain referrals from February, 2014, to November, 2015, resulting in a sample of 218 military veterans. The project used promotional “flyers” and recruited from veterans-specific housing, shelters, treatment programs, and veterans’ service agencies throughout New York City to reach members of this hard-to-reach population to talk about a sensitive topic. Eligibility requirements included opioid use in the past 30 days, including POs, heroin, methadone, and/or buprenorphine. Veteran status was confirmed via a DD-214 and/or VA or veterans housing identification. This is a convenience sample of a hard-to-reach population of great interest with complex health and social integration issues.

This analysis examines data from face-to-face administration of a computer-assisted survey instrument in a private location. Written informed consent was obtained. Participants were compensated $60 for completing the assessment that lasted roughly 2 hours. All procedures were approved by the National Development and Research Institute, Inc. Institutional Review Board. A Certificate of Confidentiality (COC) has also been obtained for all study procedures and data collection issued by the US Department of Health and Human Services.

### Measures

#### Overdose risk

Participants were asked to complete the Overdose Risk Behavior Scale (ORBS), providing self-report responses to 22 questions about the number of days during the last 30 that they engaged in various opioid overdose-related behaviors. Table 1 lists the items, which were based on a broad review of the literature. ORBS includes 5 subscales of related questions covering, 1) adherence to standards for medical use, 2) alternative methods of administration; 3) using alone, 4) using drugs that were not prescribed, and 5) using substances simultaneously that are known OD risk factors. Pouget et al. [38] presents the validation of the scale. This article examines the prevalence, clusters, dimensions and covariates of these behaviors. For this analysis, items were recoded as 0—did not affirm item, 1—reported behavior on 1-14 of the past 30 days, and 2—reported behavior on at least half of the past 30 days. This ORBS scoring system has a range of scores from 0 to 44 and exhibited strong reliability (Cronbach’s α = .88).

**Table 1:** Overdose Risk Behavior Scale (ORBS) items.

On how many of the past 30 days …
*Adherence Subscale*
A1. (If has prescription) Did you take more of your opioid pain medicine than you were directed to take at one time?
A2. Did you take opioid pain medications not just to reduce pain, but for enjoyment or to get high?
A3. Did you take opioid pain medications not just to reduce your pain, but to help you sleep?
A4. Did you take opioid pain medications not just to reduce your pain, but to help you deal with anxiety, nervousness, sadness or a bad mood?"
*Alternate Administrations Subscale*
B1. Did you sniff (snort or nasally inhale) opioid pain medications?
B2. Did you crush and smoke opioid pain medications?
B3. Did you use a syringe to inject your prescribed opioid pain medicine?
*Solitary Use Subscale*
C1. Were you alone, with no other people present, while you used more of your prescribed opioids than advised?
C2. Were you alone, with no other people present, while you used heroin?
*Non-Prescribed Use Subscale*
D1. Did you take opioid pain medicine that you got from some source other than your own doctor’s prescription?
D2. Did you use heroin?
D3. Did you use methadone, either in pill or liquid form, from a clinic or any other source? (and you are not in methadone treatment)
D4. Did you inject any opioids at all?
*Concurrent Use Subscale*
E1. Did you use heroin and any opioids pain medications the same day?
E2. Did you use methadone and any opioid pain medications on the same day?
E3. Did you use buprenorphine and any prescription opioids on the same day?
E4. Did you use anti-anxiety drugs and any opioid substance at all on the same day?
E5. Did you use sleep medication and any prescription opioids on the same day?
E6. Did you drink alcohol and use any opioids at all on the same day?
E7. Did you drink alcohol, use any opioids at all, and use anti-anxiety drugs on the same day?
E8. Did you drink alcohol, use any opioids at all, and use sleep medication on the same day?
E9. Did you use cocaine, crack, amphetamine, crystal meth or any other stimulant to try to reverse?

#### Covariates

The project assessed alcohol use disorder as well as PO and heroin use disorder using the Alcohol Use Disorder and Associated Disabilities Interview Schedule-Fourth Edition (AUDADIS-IV) [39]. The AUDADIS-IV examines past 12-month experience of four types of abuse and seven symptoms of dependence such as “[In the last 12 months, did you] find that you had to use much more of a medicine or drug than you once did to get the effect you wanted?” A score of three or more criteria for dependence or one of abuse was scored as a positive screen for a disorder.

Physical pain severity and interference were analyzed as possible motivators for increased substance use using an adapted version of the Brief Pain Inventory [BPI; 40]. Participants were asked, “What was the severity of your pain at its worst during the past month? Remember, 1 is no pain at all and 10 is the worst pain imaginable.” The mean of scores regarding the worst, average and least pain was used as the pain severity scale (α = .86). Seven questions regarding interference of pain on life activities such as “In the past 30 days, how much has your pain interfered with your normal daily activities (including work and housework)? Remember, 0 indicates no interference at all and 10 indicates complete interference.” The scores were averaged to form a pain interference scale (α = .95).

Several measures were used to assess aspects of mental health including stress, resilience, and satisfaction. The brief Perceived Stress Scale (PSS) has four questions including “In the past 30 days, how often have you felt difficulties were piling up so high that you could not overcome them?”[41]. Responses ranging from 0 (never) to 4 (very often) were summed to provide a stress scale with scores from 0 to 16 (α = .70). The Connor-Davidson Resilience Scale (CD-RISC) includes 25 question such as “I can deal with whatever comes my way?” [42]. Responses ranging from 0 (not true at all) to 4 (True nearly all of the time) were summed to provide a resilience scale (α = .94) with a range of scores from 0 to 100. A modified Satisfaction With Life Scale (SWLS) with 5 statements for endorsement, such as “In most ways my life is close to my ideal” was included [43]. Responses were coded on a five-point scale ranging from 1 (strongly disagree) to 5 (strongly agree), as opposed to the standard 7-point scale for the SWLS. Response were summed to provide a life satisfaction scale (α = .82) with a range of scores from 5 to 25.

### Data analysis

#### Latent Class Analysis (LCA)

The LCA STATA plugin was used to identify groups of participants with similar risk profiles based on the 22 ORBS items [44]. Each ORBS items was coded as a pair of binary variables distinguishing behaviors committed occasionally (at least once in the past 30 days) and those committed frequently (on at least half the previous 30 days). The project estimated models with two through seven groups. The selection of the number of groups involves informed judgement. No single standardized criterion exists for LCA. Criteria for selection include diminished return on various measures of fit and interpretability. The 5-group model was selected because it exhibited strong improvement in log-likelihood (-2758.8) over models with fewer groups (-3157.5 to -2874.7), had the lowest Akaike information criterion (AIC of 3841.2 versus 3844.2–4368.6), and the groups were highly interpretable.

LCA calculates base probabilities of group membership and conditional probabilities of responses to each of the criterion variables given membership in each group. These estimates were used to assign each participant to the group that best characterizes their ORBS responses. Then the variation in demographic and mental health characteristics was estimated and statistical significance assessed using conventional tests: ANOVA for continuous variables and χ^2^ for categorical variables.

#### Factor analysis

The LCA results indicated that there were several groups that differed primarily by frequency of risk behaviors rather than by their unique constellations of risk behaviors. Factor analysis using a varimax rotation was employed to further explore how participants varied across different groups of variables. This analysis was completely separate from the LCA. Principal components identified that six eigenvalues exceeded the conventional cutpoint of 1.0. However, closer analysis revealed that three of the factors lacked meaningful interpretation. Consequently, 4- and 3-factor models were estimated and the 3-factor model was selected as providing the clearest data summary.

## Results

### Sample characteristics

Table 2 presents the sample characteristics. Most participants were male. Most were Black. About one-fifth were Hispanic. The average age was 37. Less than half the participants were stably housed in a private or publicly-funded apartment or house; others were homeless and living in a shelter or elsewhere. Just over one-third were either working or in school. Less than one fifth were living with a partner by either marriage or cohabitation. Other statistics not shown are that most had served in the Army (61%), 41% had a last tour in the Middle East as part of U.S. Operation Enduring Freedom (OEF), 24% as part of Operation Iraqi Freedom or Operation New Dawn (OIF/OND), and the average time since separation from the military was 7.5 years.

**Table 2:** Sample characteristics.

Characteristic	% or mean	St. Dev. of mean
Female	16.1	
Black	70.9	
Hispanic	20.8	
Age (mean)	37.1	(9.7)
Stable housing	49.3	
Any college	57.6	
Employed or in school	36.3	
Living with partner	18.0	
Active duty	80.2	
Honorable discharge	66.8	
Alcohol use disorder	61.2	
PO use disorder	50.9	
Heroin use disorder	25.2	
MH treatment ever	56.5	
Alcohol treatment ever	24.5	
Drug treatment ever	32.7	
PTSD treatment ever	32.9	
TBI treatment ever	8.3	
Depression treatment ever	50.5	
Sleep treatment ever	39.4	
Anxiety treatment ever	41.7	
MH treatment in past month	28.2	
Pain severity (mean)	5.2	(2.2)
Pain interference (mean)	4.5	(2.6)
Perceived stress (mean)	7.9	(3.0)
Resilience (mean)	68.6	(17.4)
Satisfaction with life (mean)	13.6	(5.2)

### Overdose risk groups

Table 3 presents the prevalence of each risk behavior and the results of LCA. On average, participants affirmed engaging in 7.4 risky behaviors. Of note, the standard deviation was high—nearly as high as the mean. The most common behaviors affirmed by 40% or more of the sample were occasionally using POs for symptoms other than pain management including to get high, help sleep and combat anxiety; using more POs than advised when alone; using POs that were not prescribed to them; and drinking while taking POs. The question regarding whether participants used more POs than directed was asked only of those with a current prescription. The question regarding using more than advised (when alone) is based on the participant’s subjective assessment.

**Table 3:** Five opioid overdose risk groups among veterans (Latent Class Analysis).

	Total	Opioid Risk Group				
		Lower Risk	Occasional Non-adherents	Frequent Non-adherents	Occasional Heroin Users	Frequent Heroin Users
Probability	100%	50.0%	20.5%	7.4%	15.2%	6.9%
Count	218	111	43	16	33	15
Mean ORBS Score	7.4	2.4	9.0	15.2	11.6	23.3
(SD of ORBS Score)	(7.0)	(2.0)	(3.1)	(3.2)	(5.0)	(6.1)
***Occasional behaviors*:** ***Percent of participants by group that affirmed engaging in each behavior on at least one of the past 30 days (values above 0*.*50 are shown in bold)***						
used more than directed	.25	0.14	0.47	0.25	0.24	0.47
used to get high	.45	0.24	0.49	**1.00**	**0.59**	**1.00**
used to get to sleep	.50	0.33	**0.71**	**0.94**	0.39	**0.93**
used to deal with anxiety	.42	0.15	**0.65**	**1.00**	0.47	**0.93**
Sniffed	.14	0.01	0.16	0.06	0.36	**0.60**
Smoked	.06	0.00	0.04	0.00	0.21	0.20
Injected	.07	0.00	0.00	0.00	0.18	**0.60**
used more PO alone	.45	0.21	**0.69**	**0.75**	**0.56**	**1.00**
used heroin alone	.22	0.04	0.00	0.19	**0.74**	**1.00**
POs not prescribed	.53	0.39	0.49	**1.00**	**0.65**	**0.93**
heroin	.28	0.11	0.08	0.00	**0.91**	**0.93**
methadone not prescribed	.15	0.03	0.11	0.07	0.48	0.47
inject any opioids	.11	0.00	0.00	0.00	0.45	**0.60**
PO + heroin	.17	0.03	0.07	0.00	**0.60**	**0.73**
PO + methadone	.09	0.00	0.04	0.07	0.36	0.33
PO + buprenorphine	.05	0.01	0.02	0.00	0.15	0.20
PO + anxiolytics	.29	0.03	**0.63**	**0.56**	0.48	**0.53**
PO + sleep medicine	.25	0.03	**0.63**	**0.50**	0.32	0.33
opioids + alcohol	.45	0.22	**0.70**	**0.94**	**0.52**	**0.67**
opioids + alcohol + anxiety	.22	0.00	**0.51**	**0.62**	0.27	0.33
opioids + alcohol + sleep	.22	0.01	0.39	**0.63**	0.37	0.40
stimulant to reverse	.18	0.03	0.08	0.44	0.47	**0.60**
***Frequent behaviors*:** ***Percent of participants by group that affirmed engaging in each behavior on half or more (15+) of the past 30 days (values above 0*.*50 are shown in bold)***						
used more than directed	.06	0.04	0.11	0.12	0.00	0.20
used to get high	.16	0.06	0.00	**0.93**	0.00	**0.80**
used to get to sleep	.18	0.05	0.18	**0.87**	0.00	**0.73**
used to deal with anxiety	.17	0.04	0.09	**0.87**	0.00	**0.93**
sniffed	.04	0.00	0.00	0.06	0.09	0.33
smoked	.01	0.00	0.00	0.00	0.00	0.13
injected	.02	0.00	0.00	0.00	0.00	0.33
used more PO alone	.17	0.01	0.24	**0.56**	0.09	**0.93**
used heroin alone	.07	0.00	0.00	0.12	0.09	**0.67**
POs not prescribed	.17	0.06	0.09	**0.69**	0.12	**0.80**
heroin	.12	0.01	0.04	0.00	0.39	**0.67**
methadone not prescribed	.07	0.03	0.04	0.00	0.24	0.13
inject any opioids	.07	0.00	0.00	0.00	0.21	**0.53**
PO + heroin	.06	0.00	0.04	0.00	0.09	0.47
PO + methadone	.03	0.00	0.00	0.00	0.15	0.13
PO + buprenorphine	.01	0.00	0.00	0.00	0.00	0.20
PO + anxiolytics	.12	0.00	0.38	0.37	0.00	0.20
PO + sleep medicine	.09	0.01	0.29	0.19	0.00	0.20
opioids + alcohol	.14	0.03	0.12	**0.75**	0.12	0.40
opioids + alcohol + anxiety	.06	0.00	0.07	0.31	0.09	0.13
opioids + alcohol + sleep	.06	0.00	0.09	0.25	0.03	0.20
stimulant to reverse	.04	0.01	0.05	0.06	0.00	0.33

**Fig 1:**
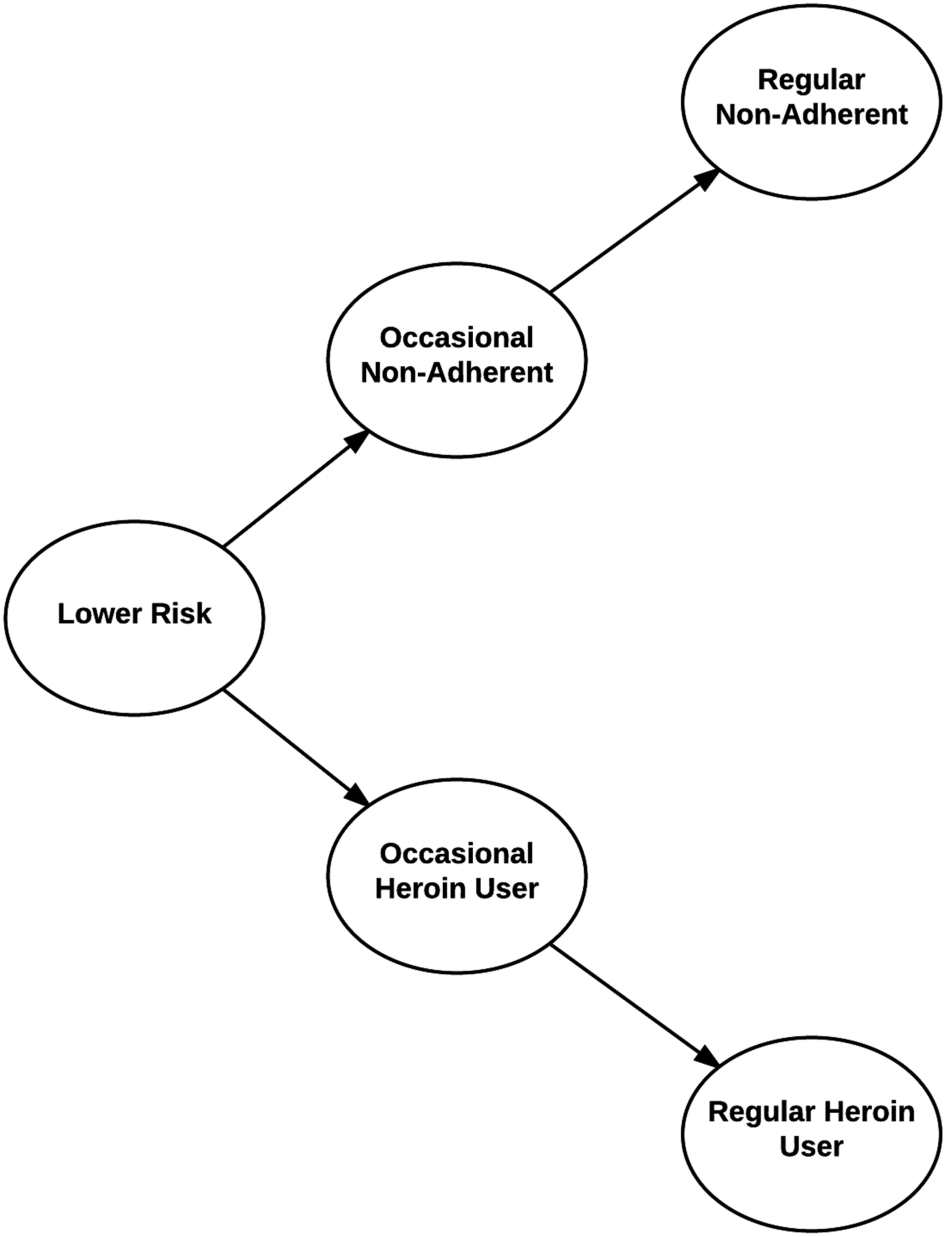
Relationship between risky behavior clusters and factors.

Group names for the LCA solution were based on the conventional criterion of identifying those items with a conditional probability of 0.50 or greater (shown in bold). The most common group had no such risk factors and was therefore named the lower risk PO users. This group had a lower risk profile than the other groups, although there were some risk behaviors that were still quite common including occasionally using POs that were not prescribed, and using POs to help sleep. The next two groups formed a nested hierarchy. Both groups of risk behaviors were primarily associated with non-adherence to prescribed use of POs. The last two groups behaviors involved heroin use and also formed a nested hierarchy. Fig 1 provides a graphical representation of the relationship across groups. The occasional non-adherents on average affirmed many more risk behaviors (9) than the lower risk PO users (2). They most commonly affirmed sometimes using POs to help sleep and combat anxiety, using more than advised when alone, and mixing POs with anxiolytics, sleep medication, and alcohol. The frequent non-adherents on average affirmed even more items (15) including those affirmed by the occasional non-adherents as well as occasionally using POs to get high and using POs not prescribed to them. The frequent non-adherents also affirmed participating in several behaviors on more than half of the past 30 days including using POs to get high, help sleep and combat anxiety, using more than advised when alone, using POs not prescribed to them, and drinking while taking POs.

The occasional heroin users affirmed 12 risk behaviors on average including some of the occasional non-adherent behaviors (use to get high, use more POs than advised when alone, used POs not prescribed to them, drank while using POs) as well as occasional behaviors associated with heroin use (used heroin, used heroin when alone, used POs and heroin on the same day). The frequent heroin users affirmed 23 risk behaviors on average including the same occasional non-adherent behaviors and several more (used POs to help sleep and combat anxiety, used POs on the same day as anxiolytics). They also affirmed occasionally engaging in several more heroin-related behaviors including injecting POs, injecting opioids, and using stimulants to reverse the effects of opioids. Additionally, they affirmed sniffing POs. The frequent heroin users also affirmed frequently engaging in several heroin-related behaviors (using heroin, using heroin alone, injecting opioids) and several non-adherent behaviors (used to get high, sleep, combat anxiety, used more than advised when alone, used POs not prescribed to them).

### Dimensions of risk behaviors

Table 4 presents the factor analysis of the ORBS items. The first dimension identifies common non-adherent behaviors (using POs to get high, sleep, combat anxiety, and using POs not prescribed to them). The second identifies dimensions of polydrug use, combining POs with anxiolytics and sleep medication. These dimensions characterize the difference between lower risk, occasional non-adherent, and frequent non-adherent PO users as illustrated in Fig 1. The third dimension identifies items associated with heroin use (using heroin, using heroin alone, injecting, injecting any opioid, using methadone not prescribed to them, and using methadone and POs on the same day) that distinguish lower risk PO users from occasional and frequent heroin users. Heroin users are also distinguished from lower risk PO users in their affirmation of non-adherent behaviors but less in their affirmation of polydrug use.

**Table 4:** Three dimensions of opioid risk behaviors among veterans (Factor Analysis).

	Factor loading by Dimension		
	Non-adherent use	Poly-Drug Use	Heroin Use
used more than directed	.200	.369	-.022
used to get high	**.770**	.141	.184
used to get to sleep	**.569**	.263	.002
used to deal with anxiety	**.690**	.335	.146
Sniffed	.391	.223	.489
Smoked	.335	.251	.410
Injected	.199	-.035	**.661**
used more PO alone	.473	.327	.195
used heroin alone	.239	.010	**.794**
POs not prescribed	**.725**	-.001	.142
heroin	.151	-.117	**.778**
methadone not prescribed	-.176	.275	**.634**
inject any opioids	.018	.003	**.747**
PO + heroin	.307	-.039	**.711**
PO + methadone	-.077	.274	**.616**
PO + buprenorphine	.096	.250	.440
PO + anxiolytics	.097	**.657**	.208
PO + sleep medicine	.067	**.740**	.023
opioids + alcohol	.420	.495	.050
opioids + alcohol + anxiety	.232	**.811**	.046
opioids + alcohol + sleep	.186	**.732**	.154
stimulant to reverse	.360	.304	.462

### Covariates of overdose behavior risk group

Table 5 presents the variation in demographics and mental health across the five risk groups. Female and black participants were less likely to be heroin users (either occasional or frequent). The lower risk PO users were more likely to be employed or in school followed by non-adherents and then heroin users. The heroin users were less likely to have stable housing, especially frequent heroin users. Lower risk PO users were the least likely to screen positive for alcohol, PO and heroin use disorder. Non-adherents were highly likely to screen positive for alcohol and PO use disorder. Heroin users were highly likely to screen positive for all three. Lower risk PO users were the least likely to report having been treated for mental health, alcohol or drug problems. This pattern tended to hold with regard to having had mental health treatment for PTSD, depression, trouble sleeping, and anxiety but not traumatic brain injury (TBI). Current mental health treatment was highest for occasional non-adherents and lowest for frequent non-adherents. We do not have a clear explanation for this finding.

**Table 5:** Sample characteristics and variation across five opioid overdose risk groups.

Characteristic	Percentage (or Mean) by Risk Group				
	Lower Risk	Occasional Non-adherents	Frequent Non-adherents	Occasional Heroin Users	Frequent Heroin Users
Female*	22	21	6	3	0
Black**	82	66	69	53	47
Hispanic	17	23	13	27	33
Age (mean)	37.1	35.1	35.7	39.6	38.8
Stable housing**	56	56	56	30	13
Any college	59	65	73	45	40
Employed or in school**	50	23	38	15	14
Living with partner	20	19	19	12	13
Active duty	82	65	88	85	93
Honorable discharge	72	65	38	67	67
Alcohol use disorder**	49	78	67	67	80
PO use disorder**	32	70	88	54	87
Heroin use disorder**	11	12	0	49	80
MH treatment ever**	37	74	81	76	80
Alcohol treatment ever*	15	28	38	39	36
Drug treatment ever**	21	30	44	64	43
PTSD treatment ever**	21	53	38	36	47
TBI treatment ever	9	9	6	6	7
Depression treatment ever**	33	70	62	67	73
Sleep treatment ever**	27	58	44	52	47
Anxiety treatment ever**	25	67	50	52	60
MH treatment in past month**	21	51	12	27	33
Pain severity (mean)**	4.7	5.8	5.9	4.8	6.5
Pain interference (mean)**	3.8	5.4	5.9	4.2	5.9
Perceived stress (mean)	8.4	7.2	7.4	8.2	7.0
Resilience (mean)*	71.8	68.6	64.4	64.9	58.5
Satisfaction with life (mean)**	15.0	12.9	10.3	11.8	11.9

* Difference across groups significant at the α = .05 level.

**Difference across groups significant at the α = .01 level.

Pain severity and interference was substantial for all five groups but lowest for the lower risk PO users and occasional heroin users. Non-adherents (both occasional and frequent) had higher rates and regular heroin users had the highest. Resilience was highest for the lower risk PO users with an average score (72) approaching the average for a general population sample (80) [45]. Resilience declined across the frequency of non-adherence groups (from lower risk to occasional non-adherent and regular non-adherent) and frequency of heroin use groups. The lowest average (58) among frequent heroin users approached the average for a group of PTSD patients. Life satisfaction was highest among the lower risk PO users with a mean score of 15 corresponding to an average score of 3, “neither satisfied nor dissatisfied.” Other groups scored lower, in the “somewhat dissatisfied” range.

## Discussion

### Key findings

When creating ORBS, we identified 5 domains of behaviors to serve as subscales. However, empirical analysis with study participants indicated two major dimensions of variation were most common: non-adherence and heroin use. These domains emerged both as dimensions from factor analysis and as clusters using latent class analysis, affirming the centrality of this finding. Those with higher ORBS scores (not in the lower risk PO user group) were also more likely to screen positive for alcohol and substance use disorders, reported greater self-medicating opioid use to relieve anxiety, reported greater problems with physical pain, were more likely to have had mental health, alcohol and drug treatment, and were less likely to be employed or in school. Heroin users also were less likely to report stable housing.

### Implications

Our results suggest that opioid overdose risk typologies are heavily grounded in contextual factors. Although causal directionality is beyond the reach of this study, results demonstrate the comorbid conditions among the greater and more frequent risk types that have led researchers to talk of drug and sexually transmitted infection “syndemics,” [46–50] involving multiple forms of overlapping social, economic, and (in the case of combat veterans) physical disadvantage. As with analyses of substance abuse among Vietnam-era veterans that showed a strong social and environmental basis for heroin use [51–53], our analysis demonstrates that higher levels of opioid overdose risk are frequently related to experiences of both psychological and physiological pain and distress, homelessness and disconnection from positive social and economic supports like schooling and gainful employment. Typologies of the sort identified here thus reinforce the need for patient management that seeks a more holistic understanding of individual health care and substance abuse treatment needs. Researchers working with veteran and substance-using populations have long argued the value of “wrap-around” interventions [54–56] and forms of outreach that coordinate different healthcare and social service modalities through an active dialogue among practitioners about the unique needs of individuals. More recently this approach has been reinvigorated through advances in the patient-centered medical home model [57–60], an approach to health care that involves both dialogue among care and service providers and the central role of a patient care coordinator [61, 62], which in some instances involves peers who share critical life experiences with patients [63–65]. These approaches address the key findings emerging from our analysis in their capacity to address the complex and multifaceted health challenges that opioid users at high risk of overdose disproportionately face. Remediation efforts should be informed by a recognition that that the factors characterizing those at greatest risk are rarely reducible to psychological motivation to “get high” or low resilience to psychosocial stress. Interventions and forms of treatment that sensitively address the ways in which pleasure is entangled with the pain related to coping challenges, injury, social marginality, and trauma stand to make considerable advances in the treatment of disadvantaged and at-risk populations, like veterans, whose substance abuse cannot productively be treated in isolation from the array of psychological and sociostructural challenges so many face.

### Study limitations

This is the first study using the ORBS scale to better understand overdose risks among a sample of post 9/11 veterans, and findings should not be generalized beyond enlisted urban veterans living in NYC in the absence of other research replicating these analyses with other populations. Moreover, the estimates provided in this paper should be interpreted cautiously given that the data are self-reports from a convenience sample of a very particular population. The data are not representative of all opioid users, all veterans, nor even all veterans using opioids. This paper uses cross-sectional data only and thus cannot illuminate the sequencing and possible causal relationships among opioid-using veterans’ life challenges.

### Future research

Future research examining typologies of overdose risk needs to examine paths and covariates from lower risk PO use, to greater engagement in non-adherent behaviors and the possibility of other non-prescribed opioids including heroin. Longitudinal analyses, potentially using related techniques like latent transition analysis, should study paths from lower and less frequent categories of risk behavior to higher ones (and vice versa) to better understand the precipitants of risk behavioral change (in either direction) and to learn to better intervene at important turning points and life transitions.
